# Inflammation as a Modulator of Host Susceptibility to Pulmonary Influenza, Pneumococcal, and Co-Infections

**DOI:** 10.3389/fimmu.2020.00105

**Published:** 2020-02-11

**Authors:** Elizabeth R. Aguilera, Laurel L. Lenz

**Affiliations:** Department of Immunology and Microbiology, University of Colorado School of Medicine, Aurora, CO, United States

**Keywords:** pulmonary inflammation, viral infection, bacterial infection, innate immunity, down syndrome

## Abstract

Bacterial and viral pathogens are predominant causes of pulmonary infections and complications. Morbidity and mortality from these infections is increased in populations that include the elderly, infants, and individuals with genetic disorders such as Down syndrome. Immune senescence, concurrent infections, and other immune alterations occur in these susceptible populations, but the underlying mechanisms that dictate increased susceptibility to lung infections are not fully defined. Here, we review unique features of the lung as a mucosal epithelial tissue and aspects of inflammatory and immune responses in model pulmonary infections and co-infections by influenza virus and *Streptococcus pneumoniae*. In these models, lung inflammatory responses are a double-edged sword: recruitment of immune effectors is essential to eliminate bacteria and virus-infected cells, but inflammatory cytokines drive changes in the lung conducive to increased pathogen replication. Excessive accumulation of inflammatory cells also hinders lung function, possibly causing death of the host. Some animal studies have found that targeting host modulators of lung inflammatory responses has therapeutic or prophylactic effects in these infection and co-infection models. However, conflicting results from other studies suggest microbiota, sequence of colonization, or other unappreciated aspects of lung biology also play important roles in the outcome of infections. Regardless, a predisposition to excessive or aberrant inflammatory responses occurs in susceptible human populations. Hence, in appropriate contexts, modulation of inflammatory responses may prove effective for reducing the frequency or severity of pulmonary infections. However, there remain limitations in our understanding of how this might best be achieved—particularly in diverse human populations.

## Introduction

Pulmonary disease constitutes four of the ten leading causes of death in the human population [chronic obstructive pulmonary disease (COPD), lung cancers, pneumonias, and tuberculosis]^1^. Each of these conditions is also associated with inflammatory reactions. Therefore, a better understanding of lung biology and the control of inflammation in the lungs during infection has potential to substantially impact human health.

The lungs are a vital organ that facilitate efficient transfer of oxygen and carbon dioxide. Their large surface area is comprised of small terminal air sacs called alveoli. In the alveoli, a single layer of epithelial cells separates inhaled air from underlying small capillaries. Maintenance of the alveolar structure and function is thus crucial for proper functioning of the lungs. Breathing exposes the upper respiratory tract and lung alveolar surface to microbes and other environmental substances. At an average of 15 breaths each minute, more than 10,000 L of air passes over airway mucosal surfaces in the course of a day (1). Each liter of air contains hundreds of thousands or even millions of microbes, thus nasal tissues and the lung alveoli may contact upwards of 10^9^ inhaled microbes each day (2). Commensal microbes also inhabit these tissues (3). To protect the lungs from overgrowth or invasion by microbes, the upper respiratory tract is coated with a mucus layer containing antimicrobial peptides and proteins. Mucus traps many inhaled microbes, which are then cleared from the respiratory tract through the activity of ciliated cells (4). The lung luminal (environmental) alveolar surface is similarly coated with a thin layer of liquid surfactant with dissolved proteins and lipids (3, 4). This surfactant adsorbs at the air/water interface to reduce surface tension, maintain lung elasticity, and capture particles from the air. Beneath the surfactant, alveolar macrophages (AMs) patrol the apical surface of epithelial cells to engulf and remove inhaled microbes (1). Though generally effective, certain pathogens can overcome these upper and lower airway defenses. Infection by such pathogens elicits innate immune cell activation and the initiation of inflammatory responses. In this review, we focus on these innate immune players in the context of lung infections.

Bacterial and viral pathogens are common causes of human pulmonary infections and will be the focus of this review. Bacteria that commonly cause human lung infections include *Haemophilus influenzae, Staphylococcus aureus*, and *Streptococcus pneumoniae* (5). Viruses that commonly cause human lung infections include Respiratory syncytial virus (RSV) and influenza viruses (5, 6). Co-infections with these bacteria and viruses is also common and is generally associated with more severe disease and a higher incidence of mortality (5). The current review provides an update and expands on elements previously reviewed by others [e.g., (7)]. Fungal pathogens also cause lung infections and co-infections with bacteria or viruses—particularly in immunocompromised individuals and individuals with polymorphisms in innate immune detection systems (8, 9). However, due to space limitations, fungal infections will not be further discussed in this review.

In this review, we provide an overview of the events that occur when innate lung defenses are overwhelmed by viral and/or bacterial pathogens. Our focus is on innate immune players in animal models of influenza A virus (IAV) and/or *S. pneumoniae* infection, though some relevant human subject studies are also mentioned. The available data support the hypothesis that the nature and magnitude of the inflammatory response contributes to host susceptibility and thus can drive overwhelmingly severe lung infection.

## Inflammatory Responses to Pulmonary Infection

**Figure 1:**
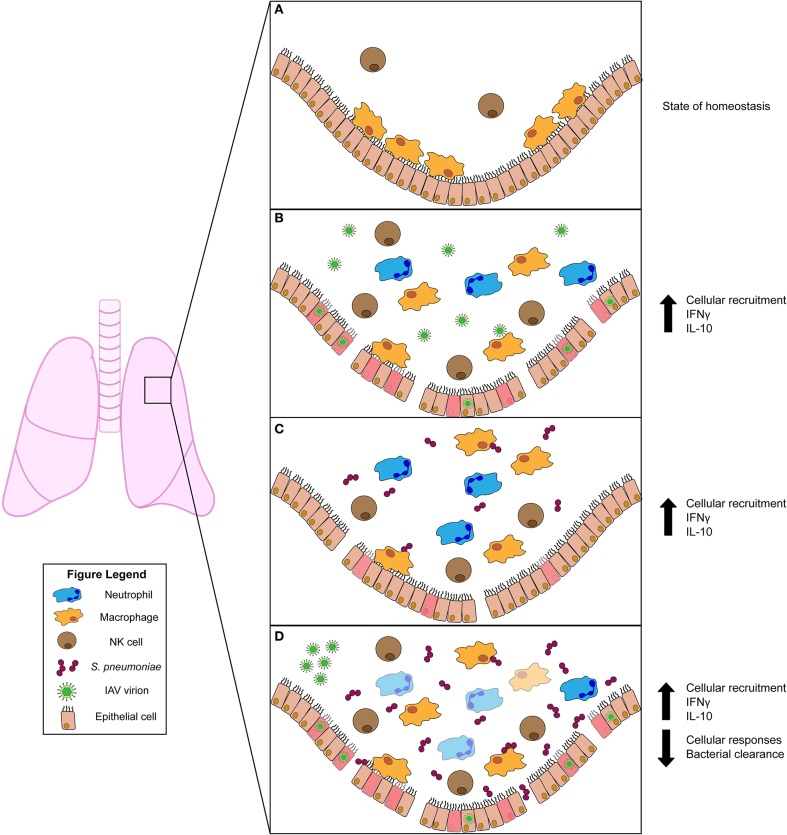
Innate immune responses to viral and/or bacterial infection in the lung. **(A)** Uninfected lung in homeostatic state harbors resident alveolar macrophages (AMs) and natural killer (NK) cells. **(B)** Influenza A virus (IAV) infection activates AMs and NK cells. Infected cells produce chemokines that recruit inflammatory phagocytic cells (such as neutrophils) to aid in clearance. The pro-inflammatory cytokine IFNγ is induced during IAV infection but has conflicting roles in animal studies. The anti-inflammatory cytokine IL-10 is produced and plays a role in regulating lung inflammation. **(C)** *Streptococcus pneumoniae* infection also stimulates activation of AM and NK cells. NK cells produce IFNγ, which plays a protective role for the host. IL-10 is also induced and modulates the influx of neutrophils into the lung. **(D)** Primary IAV infection predisposes the host to secondary *S. pneumoniae* infection. This co-infection drives increased bacterial burdens in the lung. Increased burdens correlate with diminished bacterial clearance by AMs, which is attributed to stimulation of AMs by IFNγ. Similar to *S. pneumoniae* single infection, IL-10 is also induced during co-infection. This suppresses excessive neutrophil accumulation and subsequent lung damage.

The healthy lung houses both epithelial and resident immune cell populations. Resident immune cells typically found in the healthy lung include neutrophils, monocytes, macrophages, dendritic cells, natural killer (NK), and other innate lymphocyte (ILC) populations, as well as B and T cells (10). Of these cell populations, resident AMs are most abundant (Figure 1A). When an invasive pathogen overwhelms AMs and has established an active infection, pathogen-associated molecular pattern (PAMPs) and damage-associated molecular patterns (DAMPs) can engage pattern recognition receptors (PRRs) on these cell populations (10). Ligation of PRRs leads to activation of cellular signaling pathways and the production of soluble interferons (IFNs) which drive expression of IFN-stimulated genes (ISGs) that act in a cell-intrinsic manner to prevent or limit replication of invading pathogens (10). Simultaneously, PRR ligation induces the expression and production of cytokines and chemokines which regulate the activation and recruitment of additional immune and inflammatory cell populations in the lung (10). Recognition of PAMPs by specific PRRs [e.g., toll-like receptors (TLRs) 2 and 4] thus has substantial impact on disease susceptibility and pathogen transmission. In the initial stages of infections, recruited neutrophils, monocytes, and resident AMs are considered the primary effectors of pathogen clearance (Figure 1). The influx of inflammatory myeloid and other immune cells is necessary to contain and kill invasive microbes. However, the recruitment and activities of these cells can also impair gas exchange and cause damage to the lung epithelium. Thus, fine-tuning of these responses is essential for efficient pathogen clearance and to reduce host damage associated with severe lung infections (10). When accumulation of inflammatory cells and fluid in the lung alveoli disrupts their ability to mediate gas exchange, the clinical condition known as pneumonia ensues. Pneumonias occur with increased frequency in infants, the elderly, and/or individuals who are immunocompromised or have specific genetic conditions that include Down syndrome^2^ (11). This suggests these groups have an inherent impaired ability to combat lung pathogens and/or to constrain the inflammation associated with these infections.

## Influenza Virus Infection in the Lung

One of the most common viral pathogens associated with lung disease in humans is influenza virus. Influenza virus is a segmented RNA virus belonging to the *Orthomyxoviridae* family (12). Influenza, like other RNA viruses have high genetic variability due to poor proofreading activity during replication (12). In addition, co-infection by different influenza viruses increases genetic diversity through reassortment of viral genome segments (12). These, and other factors result in antigenic and pathogenic diversity which limits effectiveness of vaccination. Consequently, influenza viruses continue to pose a serious health risk to the human population with over 3,000,000 cases and up to 650,000 deaths per year globally (average of 25,000–36,000 in the US)^3^ (6, 13).

Upon surpassing the host's initial physical and chemical barriers to infection (i.e., mucosal layer), influenza virus invades and replicates in lung epithelial cells. Mucus production offers some resistance to IAV infection, but resolution of infection requires innate immune responses (14). Indeed, viral replication in lung epithelial cells leads to cytokine production and activation of AMs that contribute to the initial control of infection (Figure 1B). Thus, mice lacking AMs had increased pulmonary viral burdens and mortality (15, 16). However, AMs are significantly reduced by 4 days after IAV infection (17, 18). Such reductions may partly be driven by cytolytic NK cells, which can recognize and target IAV-infected cells (19, 20). Indeed, activating natural cytotoxicity (NCRs) and other receptors on NK cells have been shown to bind IAV hemagglutinin proteins. When these proteins are expressed at the surface of virus-infected cells, this recognition can induce NK cells to lyse the infected target cell (19–21). Such lysis, and possibility other NK cell effector functions, contribute to early protection, since NK cell depletion increases lung damage and mortality (22). Chemokines produced by IAV-infected cells, such as CCL-1 (MCP), further protect the host by recruiting inflammatory phagocytes that help control infection (23, 24). Recruitment of neutrophils to the lung is further induced by IL-1 and plays an important role in reducing viral replication (25). Thus, effective induction of innate immune responses is critical for host resistance to IAV.

Excessive, inflammatory cell recruitment and the induction of a “cytokine storm” are hallmarks of more severe IAV infection and lung disease. These responses can be exacerbated by polymorphisms in TLRs or other PRRs. The cytokine proteins contributing to this storm include the type I IFNS, IL-1α, IL-1β, IL-6, IL-8, IL-10, IL-15, and the only type II IFN, IFNγ (25–28). Several of these cytokines appear to be beneficial to the host by contributing to host resistance. For example, mice deficient for the interferon alpha/beta receptor, IFNAR, that mediates cellular responses to type I IFNs show increased morbidity and mortality (26, 29). However, excessive production of specific cytokines drives excessive and detrimental inflammation. In particular, IFNγ can exacerbate disease severity during IAV infection. Indeed, mice lacking either expression of IFNγ or the ligand-binding subunit of its receptor, IFNGR1, showed increased survival following IAV challenge (18, 30) The increased survival in mice lacking IFNγ was shown to be associated with reduced immunopathology due to increased activity of type II innate lymphoid cells (ILC2s), which produce IL-5 and amphiregulin to promote tissue homeostasis (30). In this context, IL-5 elicited eosinophils and was required for enhanced survival of the mice lacking IFNγ. In mice lacking IFNGR1, inflammatory infiltrates and cytokine production were also reduced (18). However, in mice lacking IFNGR1 expression viral titers were reduced at 6-8 days after infection, whereas no differences in viral burden were observed up to 9 days after infection in these mice (18, 30) The reasons for this discrepancy are not clear, but could reflect the use of different IAV strains and infection timelines in these studies. Regardless, this collective data support the conclusion that IFNγ drives increased inflammation and lung damage during IAV infection (Figure 1B). Yet, other reports showed therapeutic effects of administering recombinant IFNγ early during IAV infection and demonstrated important protective roles for endogenous IFNγ against IAV during a recall infection or in mice lacking *Nos2* expression (31–33). In one study, this protection was attributed to improved NK cell responses (33). Thus, IFNγ signaling to specific cell types and/or in specific settings can have both beneficial or detrimental roles in the response to IAV infection.

Given the potential detrimental effects of pro-inflammatory cytokines such as IFNγ, it is not surprising that anti-inflammatory cytokines are also key regulators of lung damage during IAV infection. IL-10 is a key anti-inflammatory cytokine implicated during IAV infection. A variety of immune cell types can produce IL-10 and respond to this cytokine through expression of the cognate receptor. IL-10 signaling activates STAT3 and other signaling pathways to suppress production of pro-inflammatory factors such as IL-12 and IFNγ (34, 35). In the context of murine IAV infection, IL-10 is important for dampening the pro-inflammatory cytokine response and subsequent pulmonary damage to increase survival of IAV-infected mice (26) (Figure 1B). Thus, the balance of IFNγ and IL-10 responses could be a key determinant of the outcome during IAV infection, with too little IL-10 tipping the balance to excessive IFNγ, inflammation and increased disease severity.

## *Streptococcus Pneumoniae* Bacterial Infection in the Lung

Pulmonary infections are caused by both pathobiont (i.e., asymptomatically residing bacteria with pathogenic potential) and pathogenic (invasive) bacterial species, such as *S. pneumoniae*. *S. pneumoniae* (aka pneumococcus) transiently colonizes the nasopharynx asymptomatically in healthy humans with colonization rates highest in children^3^^,^^4^ However, this Gram-positive pathobiont causes ~50% of otitis media cases and is the most common cause of bacterial pneumonia in humans (36). *S. pneumoniae* can also establish invasive septicemia and meningitis with high mortality rates. In developed countries, pneumoccocal disease rates have dropped considerably in recent years due to vaccination. Nevertheless, nearly 900,000 people develop pneumococcal pneumonia each year in the United States and this remains an important cause of morbidity and mortality globally seen in immune compromised, elderly adults and particularly causing nearly 810,000 deaths in children under 5^3^^,^^4^.

Pneumonia occurs when a colonizing *S. pneumoniae* strain gains access to the lower respiratory tract. Such access is promoted by inflammatory events, which likely contribute to increased density of colonizing *S. pneumoniae*. Consistent with this, polymorphisms in PRRs has been associated with increased colonization and/or invasive infection by *S. pneumoniae* (37, 38). Inflammation is thought to reflect an increased nutrient availability following inflammation-driven epithelial damage and increased access of bacteria to adhesion receptors such as those for platelet-activating factor (PAFr) or polymeric immunoglobulin (pIgR), which are upregulated in response to inflammatory cytokines (39–41). Inflammation thus increases the density of *S. pneumoniae* in the nasopharynx and thus provides an opportunity for increased aerosolization of bacteria into the lungs (for host-derived pneumonia) and environment (for transmission). These or other effects of inflammation may partly explain the increased incidence of pneumococcal pneumonia in individuals with a primary respiratory viral infection, elderly individuals, or other populations (see further information below) (42).

Despite the evidence that aspects of inflammation promote *S. pneumoniae* colonization, murine infection models have demonstrated protective roles for certain inflammation-associated responses. Two studies reported that the type I IFN response protects mice from colonization and invasive infection following intranasal infection by a serotype 2 strain of *S. pneumoniae* (43, 44). However, a third report using the different bacterial serotype 3 strain correlated type I IFNs with increased lung bacterial burdens (29). Whether these differing results indicate distinct roles for type I IFNs in protection of distinct tissues or reflect use of distinct *S. pneumoniae* isolates remains unclear. IFNγ also appears to protect mice against pulmonary *S. pneumoniae*. Early work found that mice lacking IFNγ were more susceptible to bacteremia and mortality following intranasal infection (45). Treatment with IL-12 was subsequently shown to induce NK cell production of IFNγ and protect mice against pulmonary *S. pneumoniae* (46). However, the overall impact of NK cells in this setting may not be beneficial as NK cell depletion lowered lung bacterial burdens in infected *Scid* mice with no effect on burdens in controls (47). NK cell depletion likewise reduced survival of mice infected systemically with another streptococcus strain, *S. suis* (48). Yet, a more recent study using a genetic diptheria toxin (DT)-based approach to deplete NKp46+ NK cells found that this manipulation reduced mouse survival following pulmonary *S. pneumoniae* infection (49). Effects of the DT-induced NK cell depletion on bacterial burdens was not reported in the latter study, but the authors showed a transfer of wildtype NK cells improved survival in mice lacking the four-and-a-half LIM-only protein 2 (FHL2) significantly better than transfer of IFNγ-deficient NK cells. Though NK cell specific IFNγ improved survival in this setting, the impact of NK cell IFNγ in wildtype mice is not yet clear. Overall, IFNγ appears to play important roles during *S. pneumoniae* infection (Figure 1C).

In humans and in murine models, vaccination against pneumococcal capsular polysaccharides or killed bacteria reduces colonization and transmission of *S. pneumoniae*. In mice, vaccine-induced protection was shown to be mediated by antibody or T cell immune responses (50, 51). Together with complement, opsonizing antibodies increase the ability of neutrophils and other phagocytes to engulf and kill encapsulated pneumococci. Consistent with the importance of neutrophils, IL-17 production and neutrophil recruitment to the lungs reduce bacterial burdens (52). Moreover, protection in IL-12 treated mice correlates with increased neutrophil recruitment or survival in *S. pneumoniae*-infected lungs (46). Nevertheless, excessive neutrophil recruitment can damage lung function and increase mortality. IL-10 has been shown to dampen the influx of these inflammatory cells, as well as production of pro-inflammatory cytokines such as TNF-α, to reduce tissue damage and mortality during infection (53, 54) (Figure 1C). Supporting the interpretation that this is a key role for IL-10, mortality was increased in mice deficient for IL-10 despite reduced bacterial burdens in the lung and reduced bacterial dissemination (53). Thus, therapeutic strategies that mimic or induce IL-10 may reduce damage to lungs or other vital host tissues, though at risk of increasing bacterial burdens.

## Viral-Bacterial Co-Infection in the Lung

Influenza virus infection predisposes the host to severe disease outcomes during co-infection with *S. pneumoniae*. In this context, viral infection appears to both increase the incidence and the severity of secondary bacterial infections clinically. They are associated with high morbidity and mortality in the context of seasonal flu and were a major correlate of death during 1918 Spanish Flu and 2009 H1N1 pandemics (55–57).

A number of studies have modeled IAV/*S. pneumoniae* co-infection in mice. Importantly, IAV enhances susceptibility to multiple *S. pneumoniae* serotypes with more virulent strains exhibiting the highest susceptibility (58). Results of these studies suggest diverse mechanisms contribute to the enhanced susceptibility to secondary bacterial challenge following IAV infection. These mechanisms likely include damage to the lung epithelial barrier, which can permit increased bacterial crossing of the epithelium and may both increase nutrient availability and expose host adhesions such as PAFr or pIgR to increase bacterial numbers (59–61) (Figure 1D). A recent study additionally proposed that viral adherence to the *S. pneumoniae* surface promotes adhesion to respiratory epithelia (62). Other possible detrimental effects of IAV infection on airway physiology include altered mucus production, reduced ciliary beating and alteration of the host microbiome (63–66). Albeit, there have been conflicting results regarding the impact of influenza infection on the respiratory tract microbiome (67, 68). Yet, both murine and recent human studies agree that an initial influenza (or live-attenuated vaccine) exposure increases susceptibility to secondary (or colonizing) *S. pneumoniae* (69, 70).

IAV-driven alteration of lung immune defenses have also been implicated in increased susceptibility to *S. pneumoniae*. Susceptibility and severity of secondary bacterial infections might be impacted by TNF and IL-1β production, which increase expression of *S. pneumoniae* adhesion receptors such as PAFr and pIgR. However, in a mouse model system, pneumococci administered 7 days after IAV (when reductions in viral burdens were first observed) induced less TNF and IL1β compared to non-IAV-infected mice (71). This study attributed increased susceptibility to an impaired early bacterial clearance from the lung by AMs (71). Following IAV infection, these and other phagocytes showed reduced effectiveness at engulfing bacteria that correlated with the onset of T cell-dependent IFNγ production. Further, burdens of *S. pneumoniae* at 4 h after infection (9 days after IAV) were ~50% lower in lungs of IFNγ or IFNGR1-deficient mice than in co-infected control mice. Genetic deficiency for IFNγ or IFNGR1 or neutralization of IFNγ also improved survival from secondary pneumococcal infection. These effects correlated with IFNγ-dependent reductions in staining for MARCO on lavaged CD11c+ cells. The MARCO scavenger receptor was previously implicated in the engulfment of non-opsonized *S. pneumoniae* bacteria by AMs (72). However, while IFNγ stimulation of myeloid cells has been associated with increased phagocytic and bactericidal activity of other bacteria, it has not been demonstrated that detrimental effects of IFNγ in the IAV/*S. pneumoniae* co-infection model were due to IFNγ targeting of myeloid cells (73–75). Still, there are other lines of evidence supporting the notion that suppression of myeloid cell activity is an important mechanism driving increased susceptibility in IAV-infected animals. Specifically, the increased susceptibility correlates with increases in expression of CD200R, a negative regulator of myeloid cell function (63). Additionally, co-infection induces production of anti-inflammatory IL-10 that suppresses excessive neutrophil accumulation and host resistance (76) (Figure 1D). Type I IFNs also significantly increased the bacterial burdens following secondary exposure to *S. pneumoniae*, with little to no effect on viral burdens (29). Here, IFNAR expression correlated with reduced production of chemo-attractant CXCL2 and impaired recruitment of neutrophils to the lungs. Thus, inflammatory responses elicited by IAV infection and the induction of endogenous mechanisms for dampening these responses may collectively impair myeloid cell antibacterial activity to exacerbate pneumococcal infections.

## Inflammation and Predisposition to Lung Infections

Altered or constitutive inflammatory responses are observed in elderly individuals and in individuals with genetic predispositions such as Down syndrome (DS). These responses may contribute to the high frequency and severity of IAV and *S. pn*eumoniae infections in these populations (11, 77–80). Indeed, DS appears to accelerate aging-associated cellular processes and the health phenotype of individuals with DS overlaps with that of older non-DS individuals (81). Thus, improved understanding of inflammatory responses to lung infections in the context of DS may also provide insights into the causes and possible treatments for these infections in the elderly.

In the context of DS, production of inflammatory cytokines such as TNFα, IL-1β, IL-6, IL-8, IFNα, and IFNγ were elevated in blood samples from DS vs. sibling donors following an *ex vivo* treatment with IAV (82). In this study, expression of anti-inflammatory IL-10 was not altered in the DS cohort (82). However, IL-10 production was greater in DS blood cells following an *ex vivo* stimulation with *S. pneumoniae* (83). Thus, DS may predispose toward elevated IL-10 production during *S. pneumoniae* infection, which could dampen myeloid cell antibacterial functions and contribute to elevated bacterial burdens in DS patients, similarly observed in mice (54). However, IFNγ was also found to be increased in individuals with DS at specific time points following IAV stimulation (82, 84). This correlates with a trend toward basally increased IFNγ in DS individuals. Nevertheless, elevated production of IFNγ or other cytokines could have detrimental effects on resistance as described in the murine IAV-*S. pneumoniae* co-infection studies discussed above (Figure 1). Impairment of neutrophil function has also been shown in otherwise healthy individuals with DS (85) Thus, altered inflammatory responses could contribute to susceptibility to lung infections in DS (and elderly) individuals. However, it should be noted that other non-immune mechanisms may contribute to the susceptibility in the DS (and elderly) populations. For example, in the context of DS congenital abnormalities of the respiratory tract and altered ciliary function have also been reported (86).

## Conclusions

Pulmonary infections caused by bacterial or viral pathogens are a serious clinical problem to the global human population. This clinical problem is even more concerning for specific susceptible groups including children, the elderly, and individuals with underlying genetic conditions that include Down syndrome. Co-infections of viral and bacterial pathogens can also increase susceptibility and disease severity in the broader immune-competent human population. Exacerbated or altered innate immune and inflammatory responses are characteristic of the above-mentioned susceptible groups and likely play important roles in defining disease outcome. Conflicting results and the difficulty of extrapolating from animal models of infection to human therapy remain and should be considered in the context of efforts to identify and implement specific and effective treatments. Thus, better defining the regulation of lung innate immune responses in susceptible populations and in the context of complex environmental elements (such as the microbiota) are needed to provide avenues for development of new treatments. It is also important to keep in mind the need to appropriately tune the immune and inflammatory mechanisms to minimize damage to lung tissue while ensuring adequate resistance to infections by various pathogen types.

## Author Contributions

EA and LL wrote and edited the manuscript.

### Conflict of Interest

The authors declare that the research was conducted in the absence of any commercial or financial relationships that could be construed as a potential conflict of interest.

## Footnotes

^1^https://www.who.int/news-room/fact-sheets/detail/the-top-10-causes-of-death.

^2^https://www.cdc.gov/pneumococcal/about/facts.html.

^3^https://www.who.int/news-room/fact-sheets/detail/influenza-(seasonal).

^4^https://www.who.int/en/news-room/fact-sheets/detail/pneumonia.
